# Technology-adaptable interventions for treating depression in adults with cognitive impairments: protocol for a systematic review

**DOI:** 10.1186/s13643-015-0032-4

**Published:** 2015-04-07

**Authors:** Jane Topolovec-Vranic, Yasmeen Mansoor, Naomi Ennis, David Lightfoot

**Affiliations:** St. Michael’s Hospital, 30 Bond St, Toronto, Ontario M5B 1W8 Canada; University of Toronto, 563 Spadina Crescent, Toronto, Ontario M5S 2J7 Canada; McMaster University, 1280 Main St W, Hamilton, Ontario L8S 4L8 Canada; Ryerson University, 350 Victoria Street, Toronto, Ontario M5B 2K3 Canada

**Keywords:** Cognitive impairment, Traumatic brain injury, Neurodegenerative disease, Stroke, Depression, Interventions, Technology, Non-pharmacological, Self-help, Systematic review

## Abstract

**Background:**

Depression is a common comorbidity in individuals with cognitive impairment. Those with cognitive impairments face unique challenges in receiving the benefits of many conventional therapies for depression, and may have poorer outcomes in areas such as recovery and quality of life. However, the stigmatization of mental health disorders, cost barriers and physical disabilities may prevent these individuals from seeking mental health care. An online, self-help intervention specifically developed for adults with cognitive deficits and depression may be particularly beneficial to this population. We aim to inform the design of such an intervention through a systematic review by answering the following research question: among adults with cognitive impairment (including those with acquired brain injuries or neurodegenerative diseases), which technology-amenable interventions have been shown to effectively decrease symptoms of depression? Specifically, psychotherapeutic and/or behavioural interventions that could be delivered in a self-guided, online system will be included.

**Methods:**

Comprehensive electronic searches will be conducted in MEDLINE, EMBASE, PsycINFO and CINAHL. Additional studies will be obtained through manually searching the references of relevant systematic reviews, contacting primary authors of select articles and tracking conference proceedings and trial registries. Article titles and abstracts will be screened using predefined eligibility criteria, and then judged for their amenability to the proposed self-help, technology-based intervention. The full text of those articles with selected interventions will then be screened to determine final eligibility for inclusion. Included articles will be categorized by intervention type and assessed for risk of bias using the Cochrane Effective Practice and Organization of Care Risk of Bias tool for non-randomized trials, controlled before-after studies and interrupted time series. The primary outcome will be a change in score on a validated depression scale, and adverse events will be documented as a secondary outcome. After data extraction from selected articles, pooling of data and meta-analysis will be conducted if a sufficient pool of studies with comparable methodology and quality are identified. Alternatively, plain language summaries will be developed. The quality of evidence will be assessed using the Grading of Recommendations Assessment, Development and Evaluation (GRADE) system.

**Systematic review registration:**

PROSPERO CRD42014014417

**Electronic supplementary material:**

The online version of this article (doi:10.1186/s13643-015-0032-4) contains supplementary material, which is available to authorized users.

## Background

### The link between depression and cognitive impairment

Depression is a common mental health issue faced by individuals who experience injuries to the brain and may consequently experience cognitive impairments. Such injuries may result from, for example, acquired brain injuries (e.g. traumatic brain injury (TBI), stroke) or neurodegenerative diseases (e.g. mild cognitive impairment (MCI), dementia, Alzheimer’s disease (AD)). Approximately one third of older adults with early-phase dementia experience depressive symptoms [1]. The prevalence of clinical depression is estimated at 17% in patients with AD [2], 15.7% to 44.3% in individuals with MCI [3] and 10% to 77% among TBI survivors [4,5]. Furthermore, these depressive symptoms have been shown to be associated with impaired cognitive functioning [6].

The precise relationship between depression and cognitive impairment has not yet been concretely defined. On one hand, depression may be considered a risk factor for cognitive impairment [7]. The potentially reversible cognitive decline that accompanies depression has been previously referred to as ‘pseudodementia’ [8], and has been shown to increase the likelihood of developing irreversible dementia in later life [9,10]. One mechanism that may explain this process is the idea that the altered thinking patterns in depression, such as experiencing recurring negative thoughts, may overload executive functioning and thereby contribute to cognitive deficits [11]. Conversely, it has been argued that cognitive impairment is a risk factor for depression [12]. This is based on the hypothesis that diminished cognitive capacities may alter an individual’s perception of their surroundings, leading to depressive thoughts [11,13]. Although the relationship between depression and cognitive impairment is not yet elucidated, it is apparent that the two conditions are interconnected.

Among patients with brain injuries, the combination of injury-related and psychosocial factors may also play a role in the progression of depression. Injury-related factors that may lead to depression include changes in brain neurotransmitter levels, genetic factors, neuroendocrine changes or lesions to particular brain regions [14]. Cognitive impairment may impede social interactions and the return to normal activities, resulting in a situation conducive to depression [13].

### Impact of depression in cognitively impaired adults

Untreated depression, especially in individuals with cognitive impairments, can negatively impact a wide range of outcomes. Depression can give rise to disruptive and/or aggressive behaviour, leading to increased caregiver burden, higher rates of caregiver depression and premature nursing home placement [14]. Individuals with depression are also three times less compliant in the adherence to medical regimens in chronic illness compared to those without depression [15]. Furthermore, depression may hinder cognitive recovery by exacerbating neuropsychological impairment [16,17]. Depression in individuals with TBI can lead to worse global outcomes [18], worse social functioning [19,20], lower health-related quality of life including an inability to return to work [21,22] and suicide [23]. These poor outcomes have significant societal costs: in industrialized countries like Canada and the USA, it is estimated that 2% of the population live with lifelong disabilities resulting from TBI and roughly one in four adults with TBI are unable to return to work 1 year after injury because of physical or mental disabilities [24]. As such, incorporating treatments for depression while addressing cognitive impairments may have a wide range of positive effects on outcomes for this patient population.

### The use of technology for the treatment of depression

Unfortunately, many individuals do not receive adequate treatment for mental health issues such as depression. Even when access to a mental health practitioner is available, many individuals do not seek treatment [25-27]. This may occur due to the negative stigma surrounding depression and other mental health conditions, for the preservation of privacy concerning personal matters, or because depression may not be perceived as a priority for treatment in individuals suffering from other comorbidities. Furthermore, there may be limited resources available to treat depression, particularly among those with cognitive impairments who may already be heavily utilizing the healthcare system (e.g. nursing home residents).

Technology-delivered (e.g. the internet, mobile platforms) self-help treatment approaches for depression may be more accessible for individuals as they can be used in the privacy of one’s home on their own schedule and pace, have no waitlist and may be more economical. Such tools would also overcome transportation-related obstacles faced by individuals with cognitive impairments and co-occurring injuries or physical disabilities. While internet-based programs currently exist for treating depression among general community samples, they have not been designed to consider the specific needs of individuals with cognitive impairments [28]. For example, although completion of the internet-based MoodGym training program was associated with decreased symptoms of depression among a sample of individuals who had experienced TBI, the participants reported difficulties with reading, memory and attention when using the program [29]. Such issues were reiterated in a separate study of patients with TBI who reported that they experience cognitive limitations such as reduced memory, attention, concentration, and deficits in visual acuity which would interfere with their ability to use current technology-based therapies (unpublished observations). Patient feedback regarding such therapies included simplifying instructions, allowing for sufficient breaks between and within modules, reducing the amount of text and incorporating more user-friendly layout and designs (unpublished observations). Thus, there is an opportunity and need for developing an evidence-based technology-delivered intervention for depression customized to the needs of adults experiencing cognitive impairments.

### Objective and aims of the review

Previous research has shown that currently existing internet-delivered programs are not adapted to the specific needs of individuals with cognitive impairments, particularly with respect to impaired cognitive processing (e.g. attention, memory and executive function) [29]. To address this gap, our study team is developing a new treatment approach, namely, a self-help, treatment program for patients with cognitive impairment and depression which will be delivered via technologies such as the internet or mobile platforms (e.g. telephone, tablet computers).

The objective of this systematic review is to inform the design of such an intervention by answering the following research question: among adults with cognitive impairment (including those with acquired brain injuries or neurodegenerative diseases), which technology-amenable interventions have been shown to effectively decrease symptoms of depression? Interventions that will be considered amenable to our proposed technology-delivered intervention are behavioural or psychotherapeutic interventions that can be applied to different individuals in a standardized manner using a manualized approach.

Three specific aims will be addressed in order to meet the overarching objective of this review. The first aim is to identify potential interventions that can be adapted for use with technology by patients with cognitive impairment and depression. This will be accomplished by employing broad selection criteria and being inclusive of a wide range of studies across different clinical populations. Second, we will identify which interventions have been shown to effectively treat depression in adults with cognitive impairment. This will be accomplished through a structured synthesis of the identified studies. Finally, the risk of bias and quality of evidence assessments will aide in identifying areas of strength and limitations in the existing body of literature, and we will identify potential gaps in the literature to inform future studies. Such gaps could include a lack of evidence regarding the efficacy of a particular intervention type, the efficacy of a given intervention with certain populations or with patients at varying levels of cognitive impairment, a lack of assessment of adverse events or long-term follow-up related to an intervention, a lack of research on specific outcomes (e.g. clinician diagnosed major depression versus self-reported symptoms of depression; comorbid conditions such as anxiety) and/or assessment of cost-effectiveness.

Collectively, the outputs from this review will advance health-related knowledge and health research. The results of this systematic review will advance knowledge in the area of treatment approaches for depression in individuals with cognitive impairment by identifying treatment approaches that have been shown to be effective with this population. While there exist literature reviews of interventions for depression and/or anxiety within the proposed patient populations [30-35], no review, which we are aware of, has looked at interventions that cut across various clinical populations, and focus on the issue of cognitive impairment, rather than the clinical condition. Thus, there is an opportunity to translate findings from potentially disparate conditions to each other. Additionally, by identifying gaps in the research, this review can help inform healthcare practitioners about the state of current evidence, and also help develop recommendations for future research in this field. In the long-term, this review will also inform the development of a novel technology-delivered intervention which will aim to improve the health outcomes of individuals with cognitive impairment who also live with depression.

## Methods/design

### Study registration

This protocol is registered with PROSPERO (CRD42014014417).

### Types of study designs

A systematic review will be conducted in accordance with the Preferred Reporting Items for Systematic Reviews and Meta-Analyses (PRISMA) statement recommendations [36]. As classified by the Cochrane Effective Practice and Organization of Care [37], randomized controlled trials (RCT), non-randomized controlled trials, controlled before-after designs and interrupted time series studies (including repeated measures studies) will be included in the structured analysis and synthesis. Non-comparative studies (case series) and before-after studies without multiple measurements before and after the intervention will be captured and summarized in a separate appendix, but not formally analysed. Non-RCTs will be included as 1) our scoping searches identified only a few RCTs, and 2) we do not want to exclude any potential interventions of interest. We are not aiming to identify only one, best intervention but rather the range of possible ones to consider for implementation in the planned intervention program. Articles published in the English language in a peer-reviewed journal will be included whereas abstracts, review articles, cohort designs or case series with five or less participants will be excluded.

### Types of participants

Studies will be considered for inclusion in this systematic review if they assessed adults with cognitive impairment as demonstrated using validated cognitive measures. Studies of populations with cognitive impairments due to factors other than neurodegenerative disease or acquired brain injuries (e.g. congenital disorder, fetal alcohol syndrome or developmental disability from cerebral palsy, muscular dystrophy, autism, and spina bifida with hydrocephalus) will be excluded.

### Types of interventions

Studies which assessed a behavioural/psychotherapeutic intervention or treatment will be included in the review. This may include 1) cognitive rehabilitation (e.g. memory training, cognitive stimulation therapy, cognitive training, neuropsychological training, neurorehabilitation), 2) psychotherapy (e.g. cognitive behavioural therapy, interpersonal therapy, problem-solving therapy, supportive therapy, counselling, social skills training), 3) psychoeducation, 4) exercise/physical activity and 5) others. Studies will be excluded if they assessed interventions involving the administration of a drug or procedure (e.g. acupuncture, deep brain stimulation, electroconvulsive shock therapy, sense-based therapy) which are not amenable to adaptation for delivery by internet or mobile technologies or those that did not clearly define the intervention (e.g. studies examining multi-disciplinary clinic use).

Studies that meet the aforementioned criteria will subsequently undergo a secondary screening phase in which the interventions investigated will be assessed for amenability to technology. Specifically, psychotherapeutic and/or behavioural interventions that can potentially be delivered in a self-guided, automated format will be considered amenable for delivery via technology. Specific criteria will be used to define whether an intervention is amenable to technology as follows: 1) it must follow a manual, protocol, or systemic approach in order to deliver a structured form of treatment consistently to all patients; 2) it must not rely on group discussion, group interaction, or therapeutic interaction; and 3) it must not be highly individualized or catered towards individual patients’ unique problems or life histories. Those studies considered amenable to technology will be included in the review, whereas those studies considered not amenable to technology will not be included in the review but may be referred to in the discussion. A second reviewer will confirm that a study intervention meets these secondary criteria, and any disagreements will be resolved through discussion and establishing a consensus.

Note that interventions that could be systematically customized for individual patients in terms of difficulty level will not be excluded, as this feature can be implemented into a technology-based format. Interventions in which altering behaviour is not the primary focus of treatment (e.g. cognitive training) will also be considered for inclusion. Interventions incorporating feedback will also be considered for inclusion as feedback can be automated using a technology format.

### Types of controls

Control conditions could include comparator interventions/treatments, usual care or waitlist. There will be no exclusions for comparator interventions (i.e. may or may not be amenable to technology). As indicated above, non-comparator studies (e.g. case series) or before-after studies without multiple measurements before and after the intervention will be captured in a separate appendix to be inclusive of potential interventions; however, these studies will not be used to calculate pooled estimates and will not be subject to a separate risk of bias assessment.

### Types of outcomes

Eligible studies will have assessed depression or depressive symptomatology pre- and post-intervention using a validated assessment tool. Note that depression will not need to be the primary goal of treatment in the study. A validated assessment tool could include any self-reported or interviewer-administered scale (e.g. Center for Epidemiological Studies Depression Scale [38], Beck Depression Inventory [39]) or clinician diagnosis based on standard diagnostic criteria (e.g. DSM-IV definition [40]). Secondary outcomes will include any reported adverse events associated with the interventions.

### Information sources

Searches will be conducted using the following electronic databases: PubMed including MEDLINE records, CINAHL, EMBASE and PsycINFO. In addition, reference lists of systematic reviews and relevant papers will be manually searched. The lead author on all included studies and other experts in the field will be contacted to request details of any further published or unpublished studies (i.e. in press). Proceedings of key conferences will also be tracked as well as trial registries to identify additional unpublished studies.

### Search strategy

Broad search terms will be applied to electronic databases corresponding to each of the listed inclusion criteria. Search terms related to cognitive impairment include ‘cognitive deficits’ and those relating to organic brain syndromes such as: ‘neurodegenerative disease’, ‘acquired brain injury’, ‘aging’ and ‘dementia’. In order to narrow down to articles with technology-adaptable interventions, articles with interventions utilizing drugs and procedures will be excluded by specifying exclusion terms such as ‘antidepressants’, ‘pharmacotherapy’, ‘surgery’, ‘electroconvulsive therapy’ or ‘deep brain stimulation’. Lastly, variations of the term ‘depression’ and the names of validated depression tools will be included in the search. Limits will be applied to find articles published in English and conducted on humans. Complete search strategies for each database are outlined in Table 1.

**Table 1:** **Database-specific search strategies**

**Database**	**Detailed search terms**
PubMed	((((((((((((((((((((((((‘beck depression inventory’) OR ‘zung self rating depression scale’) OR ‘patient health questionnaire 9 depression scale’) OR ‘phq 9’) OR ‘structured clinical interview for dsm’) OR ‘scid’) OR ‘hamilton rating scale for depression’) OR (‘hospital anxiety and depression scale’)) OR ‘minnesota multiphasic personality inventory’) OR ‘diagnostic interview schedule’) OR ‘brief symptom inventory’) OR ‘short form 36 health survey’) OR ‘sf 36’) OR ‘neurobehavioural functioning inventory’) OR ‘composite international diagnostic interview’) OR ‘cidi’) OR ‘present state examination’) OR ‘center for epidemiological studies depression scale’) OR ‘cesd’) OR ‘self rating scale’) OR ‘brief symptom inventory’)) AND ((((((((((((((((((((((‘cognition disorder’ OR ‘cognition disorders’ OR ‘cognition dysfunction’))) OR ((‘cognitive defect’ OR ‘cognitive defects’ OR ‘cognitive deficiencies’ OR ‘cognitive deficiency’ OR ‘cognitive deficient’ OR ‘cognitive deficit’ OR ‘cognitive deficits’ OR ‘cognitive deterioration’))) OR ‘alzheimer’) OR ‘alzheimer disease’) OR ‘brain damage’) OR ‘brain injury’) OR exp AND ‘dementia’) OR ((‘neurodegenerative’ OR ‘neurodegenerative brain diseases’ OR ‘neurodegenerative brain disorder’ OR ‘neurodegenerative brain disorders’ OR ‘neurodegenerative dementia’ OR ‘neurodegenerative dementia diseases’ OR ‘neurodegenerative disease’))) OR ((‘mini mental state’ OR ‘mini mental state exam’ OR ‘mini mental state exam mmse’ OR ‘mini mental state exam mmse score’)))) OR ‘stroke’) OR ‘cerebrovascular accident’) OR ‘cerebrovascular disorder’)) NOT (((((((‘drug therapy’) OR ‘surgery’) OR ‘drug’) OR ‘acupuncture’) OR ‘light modification’) OR ‘telestroke’))) AND depress*[Title/Abstract])) NOT ‘case report’)) NOT ‘caregiver’))) AND ((((‘therapy’) OR ‘intervention’) OR ‘psychotherapy’) OR ‘treatment’)
PubMed	(((((((((((((((((((((((((((((((‘traumatic brain injury’) OR ‘cognitive impairment’) OR ‘cognitive defect’) OR ‘cognitive deficit’) OR ((‘cognitive deficiencies’ OR ‘cognitive deficiency’))) OR ‘alzheimer’) OR ‘dementia’) OR ‘parkinson’) OR ‘neurodegenerative disease’) OR ‘mmse’) OR ‘mini mental state exam’)) AND depress*[Title/Abstract]) AND (((((‘treatment’) OR ‘intervention’) OR ‘program’) OR ‘self help’) OR ‘therapy’)) NOT ‘deep brain stimulation’) NOT ‘electroconvulsive’) NOT ‘surgery’) NOT ‘drug’) NOT ‘pharmacotherapy’) NOT ‘antidepressant’) NOT ‘caregiver’[Title/Abstract]) NOT ‘prevalence’) NOT ‘risk factors’) NOT ‘case report’) NOT ‘acupuncture’)) NOT relationship[Title/Abstract]) NOT ‘serotonin reuptake inhibitor’) NOT etiology[Title/Abstract]) NOT screening[Title/Abstract]) NOT ‘pharmacology’) NOT ‘drug therapy’ FILTER: humans
PsycINFO	1 traumatic brain injury.mp. or exp Traumatic Brain Injury/(12988)
	2 exp Cognitive Impairment/or exp Brain Damage/or exp Alzheimer’s Disease/(70020)
	3 exp Dementia/or exp Cerebrovascular Accidents/or exp Cerebral Ischemia/or Stroke.mp. (71050)
	4 neurodegenerative disease.mp. or exp Neurodegenerative Diseases/(49039)
	5 exp Aging/or exp Mini Mental State Examination/or exp Dementia with Lewy Bodies/or mmse.mp. (40902)
	6 exp Brain Damage/or exp Head Injuries/or brain injury.mp. (28035)
	7 1 or 2 or 3 or 4 or 5 or 6 (156147)
	8 therapy.mp. or exp Treatment/(509218)
	9 intervention.mp. or exp Intervention/(157539)
	10 program.mp. (137493)
	11 8 or 9 or 10 (685772)
	12 depress$.ti,ab. (182823)
	13 7 and 11 and 12 (5006)
	14 exp Electroconvulsive Shock Therapy/or exp Electroconvulsive Shock/or electroconvulsive.mp. (5620)
EMBASE	1 cognitive impairment.mp. or cognitive defect/(110884)
	2 exp brain damage/(18604)
	3 traumatic brain injury.mp. or exp brain injury/or exp traumatic brain injury/or exp head injury/(207692)
	4 neurodegenerative disease.mp. or exp degenerative disease/(375596)
	5 exp aging/or aging.mp. (412945)
	6 mmse.mp. or exp Mini Mental State Examination/(21451)
	7 exp ‘mixed depression and dementia’/or exp senile dementia/or dementia.mp. or exp dementia/or exp HIV associated dementia/(236836)
	8 1 or 2 or 3 or 4 or 5 or 6 or 7 (1094564)
	9 exp therapy/or therapy.mp. (6536263)
	10 intervention.mp. or exp intervention study/(474236)
	11 exp education program/or exp health program/or exp program impact/or exp program efficacy/or exp program evaluation/or program.mp. or exp program feasibility/or exp program effectiveness/(606821)
	12 9 or 10 or 11 (7082345)
	13 depress$.ti,ab. (378701)
	14 8 and 12 and 13 (18684)
	15 drug therapy.mp. or exp drug therapy/(1682801)
	16 antidepressant.mp. or exp antidepressant agent/(305303)
	17 exp risperidone/or exp clozapine/or exp quetiapine/or exp ziprasidone/or exp haloperidol/or exp chlorpromazine/or exp olanzapine/or exp neuroleptic agent/or exp atypical antipsychotic agent/or antipsychotic.mp. (204343)
	18 exp electroconvulsive therapy unit/or exp electroconvulsive therapy/or electroconvulsive.mp. (16851)
	19 caregiver.ti,ab. (18046)
	20 exp epidemiology/or epidemiology.mp. (2037231)
	21 risk factor.ti,ab. (163835)
	22 prevalence.ti,ab. (484953)
	23 gene.ti,ab. (1334632)
	24 etiology.ti,ab. (160945)
	25 relationship.ti,ab. (789321)
	26 validation.ti,ab. (136292)
	27 screening.ti. (126683)
	28 deep brain stimulation.mp. or exp brain depth stimulation/(24416)
	29 surgery.mp. or exp surgery/(3551586)
	30 marker.ti,ab. (317173)
	31 15 or 16 or 17 or 18 or 19 or 20 or 21 or 22 or 23 or 24 or 25 or 26 or 27 or 28 or 29 or 30 (8808822)
	32 14 not 31 (5012)
	33 case report/(1879027)
	34 32 not 33 (4829)
	35 association.ti,ab. (1074371)
	36 34 not 35 (4322)
CINAHL	( (MH ‘Brain Injuries+’) OR (MH ‘Delirium, Dementia, Amnestic, Cognitive Disorders+’) OR (MH ‘Cognition Disorders+’) OR (MH ‘Neurodegenerative Diseases+’) OR (MH ‘Aging+’) OR (MH ‘Dementia+’) OR (MH ‘Dementia, Vascular+’) OR (MH ‘Dementia, Multi-Infarct’) OR (MH ‘AIDS Dementia Complex’) OR (MH ‘Lewy Body Disease’) OR (MH ‘Dementia, Senile+’) OR (MH ‘Dementia, Presenile+’) ) AND ( ‘therapy’ OR (MH ‘Experimental Studies+’) OR ‘intervention’ OR (MH ‘Program Implementation’) OR (MH ‘Program Evaluation’) OR ‘program’ OR (MH ‘Therapeutic Trials’) OR ‘therapeutic’ ) AND AB ( (MH ‘Depression+’) OR (MH ‘Self-Rating Depression Scale’) OR (MH ‘Hamilton Rating Scale for Depression’) OR (MH ‘Geriatric Depression Scale’) OR (MH ‘Edinburgh Postnatal Depression Scale’) OR (MH ‘Death Depression Scale’) OR (MH ‘Center for Epidemiological Studies Depression Scale’) OR (MH ‘Beck Depression Inventory, Revised Edition’) ) NOT ( (MH ‘Epidemiology+’) OR (MH ‘Epidemiological Research+’) OR (MH ‘Risk Factors+’) OR (MH ‘Acupuncture+’) OR (MH ‘Surgery, Operative+’) OR (MH ‘Drug Screening Assays, Antitumor’) OR (MH ‘Drug Evaluation, Preclinical’) OR (MH ‘Drug Rehabilitation Programs+’) OR (MH ‘Drug Design’) OR (MH ‘Drug Compounding’) OR (MH ‘Antidepressive Agents+’) OR (MH ‘Antidepressive Agents, Tricyclic+’) OR (MH ‘Antidepressive Agents, Second Generation+’) OR (MH ‘Electroconvulsive Therapy’) OR (MH ‘Deep Brain Stimulation’) OR (MH ‘Case Studies’) OR (MH ‘Gene Expression’) OR (MH ‘Mutation+’) OR (MH ‘Genes+’) OR (MH ‘Drug Therapy+’) OR (MH ‘Hemodialysis+’) OR (MH ‘Prevalence’) OR (MH ‘Cross Sectional Studies’) OR (MH ‘Surveys+’))////LIMITS: English language; human; peer-reviewed; language: English; age groups: adolescent: 13 to 18 years, adult: 19 to 44 years, middle aged: 45 to 64 years, aged: 65+ years, aged, 80 and over

### Title and abstract screening

After records are identified through the electronic search, titles will be screened for relevancy. Abstract screening will be conducted in two phases. In the first phase, the abstracts of articles with relevant titles will be screened using the primary selection criteria. In the second phase, the abstracts will be more closely screened using the secondary selection criteria, which involves assessing whether interventions are amenable to technology. Articles with unclear interventions will be further assessed during the full-text-screening step.

### Full text screening

The full text of the selected articles will be screened in detail for inclusion using a piloted form (Additional file 1) that addresses each of the primary and secondary selection criteria. The results of the full-text screening will also be assessed by a second reviewer to confirm inclusion of articles, and any disagreements will be resolved through discussion and establishing a consensus.

During full-text screening, the inclusion/exclusion criteria of each study will be evaluated in detail in order to assess whether the study participants had cognitive impairment. This criterion will be met if the mean score of the study sample on a validated cognitive measure is below a published cut-off score for normal cognitive function. Diagnostic rating scales to establish the diagnosis of a cognitive disorder (e.g. dementia) will be considered as valid cognitive measures. For cognitive measures that do not have cut-off scores specified, a study will be included if the scores of participants on the cognitive measure fall at least one standard deviation below published normative scores for the appropriate age range. Studies that mentioned the use of a cognitive measure in their inclusion criteria, but did not publish the cognitive measure data from the population prior to implementation of the intervention will be excluded. The methodology of each study will be assessed in order to confirm whether a validated measure for depression was applied to the sample in the study pre- and post-intervention. Studies that mentioned the use of a depression measure but did not present the data and/or results of these measures will be excluded. The amenability of an intervention to technology will be assessed by reviewing the description of each study’s methods. Discussion between two reviewers will be used to establish a consensus regarding whether or not an intervention is amenable to technology based on the secondary selection criteria.

**Figure 1:**
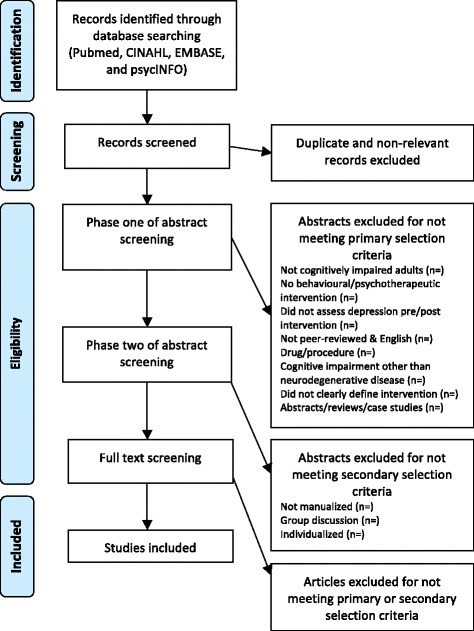
**Flowchart outlining study selection process.** The study process is comprised of four steps: identification, screening, eligibility and final inclusion. Identification of studies will be primarily through the electronic literature search. Screening will involve title screening and removal of duplicate records. Assessing eligibility will involve three steps: phase one abstract screening (applying the primary selection criteria), phase two abstract screening (applying the secondary selection criteria) and full-text screening. Studies that pass all phases of eligibility screening will be included in the review.

A flowchart outlining the full study selection process is shown in Figure 1.

### Data extraction

Data will be extracted from each article as follows: study design, details of the intervention (name, goal, format, duration, whether caregivers were involved and description of structure), details of the control condition (where applicable), sample characteristics (inclusion and exclusion criteria, indication of cognitive impairment at baseline, presence or absence of clinical depression at baseline, mean age, percentage of males and sample size) and depression outcomes (depression scale used, within-group and between-group results and results from follow-up where applicable). Notes will also be made of any key interpretations made by the authors. See Additional file 2 for the piloted data extraction form.

### Assessment of risk of bias and methodological quality

Due to the wide range of study types to be included in the review, the Cochrane Effective Practice and Organization of Care Risk of Bias Tool [41] will be used to assess for bias in RCTs, non-randomized controlled trials, controlled before-after studies and interrupted time series studies. The tool will be used to categorize each study into low, high or unclear risk of bias for the main outcome (depression score) on each of the nine standard domains (seven for interrupted time series studies), across domains and across studies as guided by the tool. Two raters will independently apply the tool to all studies and inter-rater reliability will be measured. Any discrepancies will be discussed to achieve consensus. Risk of bias tables will be generated for each study and summarized in a figure using RevMan.

### Data analyses

Each study will be summarized in a table indicating its study design, sample size, study population (clinical condition in the sample, mean age, sex), intervention (name, goal, content and structure), control/comparison groups (where applicable; their name and brief description) and the results of the study (pre- and post-scores on the depression outcome measure). The primary outcome of ‘depression score’ will be analysed as a continuous variable. Standardized mean differences (SMD) with 95% CI will be calculated as it is anticipated that studies will use different scales to measure depression symptoms. Where available, the mean change score (changes from baseline) will be reported instead of follow-up scores. The secondary outcome of ‘adverse events’ will be analysed as a dichotomous outcome and expressed as a risk ratio (RR) with 95% confidence intervals.

### Assessment of heterogeneity

Given the diverse populations and interventions that we anticipate will be included in the review, we will test the degree of heterogeneity between studies first by visually inspecting graphs between studies and then using the *χ*^2^ test and *I*^2^ statistic in RevMan. If substantial degrees of heterogeneity are found (*χ*^2^*P* < 0.1; *I*^2^ > 0.5) [42], we will pool studies using a random, rather than fixed, effect model and will explore the reasons for the heterogeneity using sub-group analyses.

### Assessment of publication bias

A funnel plot will be prepared if there are a sufficient number of studies (≥10) by plotting trial effect against standard error [43]. Possible reasons for any asymmetry will be discussed.

### Data synthesis

Studies will be categorized according to intervention type. It is anticipated that potential categories may broadly include 1) cognitive rehabilitation (e.g. memory training, cognitive stimulation therapy, cognitive training, neuropsychological training, neurorehabilitation), 2) psychotherapy (e.g. cognitive behavioural therapy, interpersonal therapy, problem-solving therapy, supportive therapy, counselling, social skills training), 3) psychoeducation, 4) exercise/physical activity and 5) others. The studies within each intervention category will then be summarized. Evidence from RCT and non-RCTs will be presented separately rather than combined [44,45]. As our review will cut across various clinical populations and include many different types of interventions, careful consideration will be given as to whether conducting a meta-analysis will be appropriate. As discussed in the EPOC resources for review authors, calculation of an average effect across studies may be meaningless if there are ‘differences in populations, interventions, comparisons or methods’ [46]. If there is a sufficient pool of studies with comparable methodology and quality, a meta-analysis will be conducted using RevMan in accordance with the statistical guidelines presented in the Cochrane Handbook for Systematic Reviews of Interventions (version 5.1.0) [42].

If a meta-analysis is deemed inappropriate, we will report plain language summaries with the ‘worksheets for preparing summary of findings tables using GRADE’ [47]. This tool will also enable us to assess the quality of evidence for each outcome with the GRADE system [48]. For each category of intervention, we will summarize the category as a whole, and any variations/adaptations to the interventions will be noted. A comparison of the similarities and differences among studies with effective versus ineffective interventions will be summarized. This step will be done to identify recurring elements of successful versus unsuccessful interventions (e.g. modifications made to a standardized treatment protocol, involvement of caregivers) and/or characteristics of studies that assessed successful versus unsuccessful interventions (e.g. with respect to sample size, quality, whether or not depression was a primary outcome measure etc.).

### Subgroup analysis

For outcome data at follow-up time points (when available), we will group the time periods as follows: baseline (0 to 3 months), medium-term (3 to 6 months) and long-term follow-up (greater than 6 months) [49]. We will conduct subgroup analyses to identify whether there are differences in depression change scores based on the duration and/or frequency of the intervention, between patients with mild/moderate versus severe cognitive impairment or across clinical populations.

### Sensitivity analysis

We will assess whether including or excluding lower quality studies or those with higher risk of bias affects the comparison between groups.

## Discussion

Several possible challenges are anticipated with this review. In order to inform the design of the planned intervention program, we aim to be comprehensive in the identification of interventions that are effective for the treatment of depression for individuals with cognitive impairment. Thus, it is possible that a large, unmanageable number of relevant articles will be identified. However, given that only eight studies of psychotherapeutic or rehabilitation interventions for depression for patients with TBI were identified by Fann et al. [17] and only three studies were identified with a non-pharmacological intervention for depression after mild TBI in a more recent meta-analysis [50], it is unlikely that the body of literature to review will be overly extensive. Note, neither of these reviews examined the issue of cognitive impairment.

A second potential challenge may arise in the inclusion/exclusion criteria related to cognitive impairment. We recognize that a large number of health-related and other (e.g. substance use) conditions/factors may result in cognitive impairment. However, we are aiming to capture interventions that have been shown to be effective in individuals with acquired brain injuries or neurodegenerative processes, our particular populations of interest. Any articles that the reviewers are uncertain about will be discussed by the study team to achieve consensus regarding its inclusion in the review.

We also anticipate that the decision to include a wide range of study designs in this review may render it difficult to effectively compare the results from studies with drastically differing methodological quality. To accommodate our aim to be inclusive of the types of interventions that exist and have been used with individuals with cognitive impairments, we will include both RCT and non-RCTs in the analyses (although separately), as well as uncontrolled studies in an Appendix. This will allow for the identification of newer interventions which may be currently only undergoing their piloting phase and have not yet had the opportunity to be assessed in the form of an RCT. Although these latter interventions are unlikely to be included in the planned intervention, they will be noted and tracked for possible future iterations of the program. Furthermore, we anticipate that assessing the risk of bias and quality of evidence will facilitate the process of placing into context the strength of evidence provided by each included article. In including a variety of study designs, we will be able to compare and contrast the interventions with the largest treatment effects and supporting strength of evidence. By doing so, we may be able to identify recurring elements of the most effective interventions (e.g. modifications made to a standardized treatment protocol, involvement of caregivers) and/or characteristics of studies that differentiated successful versus unsuccessful interventions (e.g. sample size, quality, whether or not depression was a primary outcome measure).

Another potential limitation of the review is language bias, as only studies published in English will be included in this review. This limitation will be kept in mind when interpreting the results of the review.

Our overall goal is to identify interventions that will be amenable to delivery via technologies such as the internet and/or mobile devices (e.g. smart phones, tablets, PCs). However, as technology itself is consistently changing, it is difficult to place concrete limits on whether or not an intervention is amenable to technology. We have decided to focus on developing a psychotherapeutic and/or behavioural technology-delivered intervention, as the feasibility of developing such interventions has already been demonstrated through currently available online psychotherapy interventions for depression for the general population [51-53]. Exercise therapy/physical activity interventions will also be included in the review as a growing body of literature suggests that they may be effectively delivered via technology [54-57]. Nevertheless, in the future, it may also be promising to investigate the potential for adapting other forms of therapy, such as music therapy, into a technology-based format for treating depression in both general populations and/or those with cognitive impairments.

The knowledge gained from this systematic review will be used by the research team to build the evidence base upon which we will develop the planned technology-based intervention program for depression for adults with cognitive impairments. The findings will also be disseminated through peer-review publication, which will inform clinical decision-making regarding individuals with cognitive impairment. In particular, bringing together evidence from various clinical populations with cognitive impairment may help to inform clinicians and researchers and bring to light other potential non-pharmacological treatments for exploration with patients with cognitive impairment and vice-versa.

## Additional files

Additional file 1:
**Full-text-screening form.** Each column of the form corresponds to each inclusion/exclusion criteria outlined in the methods of the study protocol. The green cells refer to inclusion criteria, whereas the red cells refer to exclusion criteria. Additional file 2:
**Data abstraction form.** The piloted form to extract full data from included studies, in which the purple cells refer to intervention details, blue cells refer to study sample characteristics, and orange cells refer to outcomes on depressive symptomatology and adverse events.
